# Analysis of Dynamic Behavior of Multiple-Stage Planetary Gear Train Used in Wind Driven Generator

**DOI:** 10.1155/2014/627045

**Published:** 2014-01-05

**Authors:** Jungang Wang, Yong Wang, Zhipu Huo

**Affiliations:** ^1^School of Mechanical Engineering, Shandong University, Jingshi Road 17923, Jinan, Shandong 250061, China; ^2^Key Laboratory of High-Efficieny and Clean Mechanical Manufacture, Ministry of Education, School of Mechanical Engineering, Shandong University, Jinan, Shandong 250061, China

## Abstract

A dynamic model of multiple-stage planetary gear train composed of a two-stage planetary gear train and a one-stage parallel axis gear is proposed to be used in wind driven generator to analyze the influence of revolution speed and mesh error on dynamic load sharing characteristic based on the lumped parameter theory. Dynamic equation of the model is solved using numerical method to analyze the uniform load distribution of the system. It is shown that the load sharing property of the system is significantly affected by mesh error and rotational speed; load sharing coefficient and change rate of internal and external meshing of the system are of obvious difference from each other. The study provides useful theoretical guideline for the design of the multiple-stage planetary gear train of wind driven generator.

## 1. Introduction

The planetary gear train, having advantages of large transmission ratio, simple construction, compactness, and smooth running, has been widely applied in many machines. In spite of these advantages, planetary gears may have undesirable dynamic behavior resulting in much noise, vibration, and other unacceptable performances. A number of papers have been published on planetary gear dynamics which comprise lumped-parameter models and deformable or hybrid models of varying complexity [1–7]. Modal analyses were performed by Lin and Parker [8] and Bodas et al. [9–13], who emphasized the structured modal properties of single-stage drives and showed that only planet, rotational, and translational modes could exist. It is important to understand the fundamental cause of the unequal load sharing behavior in planetary transmissions. The input torque applied should theoretically be shared by each planet in an *n*-planet; that is, each sun-pinion-ring path should carry 1/*n* of the total torque. However, in actual transmissions, there is unequal load sharing between the parallel paths. Du et al. [14] found the deformation compatibility equations and the torque balance equations of the 2*K*-*H*-type planetary transmission system based on the characteristic that the system is composed of a closed loop of power flow. In consideration of the manufacturing error, assembly error, and float of the parts, the load sharing coefficient of each planetary gear was calculated by using the theory of equivalent mesh error and equivalent mesh stiffness. Gu and Velex [15] presented an original lumped parameter model of planetary gears to account for planet position errors and simulate their contribution to the dynamic load sharing amongst the planets. Singh [16] developed the concept of an epicyclic load sharing map to describe the load sharing characteristics of every epicyclic gear set at any positional error and torque level. A comprehensive experimental study [17] was conducted to study the load sharing behavior of a family of epicyclical gear sets with varying number of planets. Experiments were conducted at several error and torque levels. The results clearly showed the influence of positional errors and that the sensitivity of the epicyclical gear set increased as the number of planets increased. A physical explanation [18] has been provided for the load sharing behavior. Load required to produce the needed deformation is the cause of the unequal load sharing. This explains the effectiveness of system float in reducing the load sharing inequality. Lu et al. [19] presented a calculative model for single-stage planetary gear with the dynamic way to study the load sharing behavior of each planetary gear and the relationship between error and load sharing was analyzed. Ye et al. [20] built an analytical model for NGW planetary gear train with unequal modulus and pressure angles and analyzed the load sharing behavior of each planet.

Although the references available focused on different fields, most of them established mathematical model of one-stage planetary gear train. Dynamic model of multiple-stage planetary gear train is limitedly reported. Few reports about dynamic model of multiple-stage planetary gear train composed of two-stage planetary gear train and one-stage parallel axis and its dynamic load sharing characteristics are concerned.

In this study, a transmission scheme of load-split two-stage planetary gear used in wind driven generator is proposed. Transmission ratio of the planetary gear train is obtained, as well as the relationship between transmission ratio and characteristic parameter of planetary gear train, according to conversion mechanism method and general relationship among the speed of each unit in planetary gear train. Dynamic model of load-split multiple-stage gear train composed of a two-stage planetary gear train and a one-stage parallel axis gear is established on the basis of lumped parameter theory and influence of revolution speed and mesh error on dynamic load sharing characteristic of the system is analyzed.

## 2. Load-Split Two-Stage Planetary Gear Train

### 2.1. Kinematic Scheme

**Figure 1:**
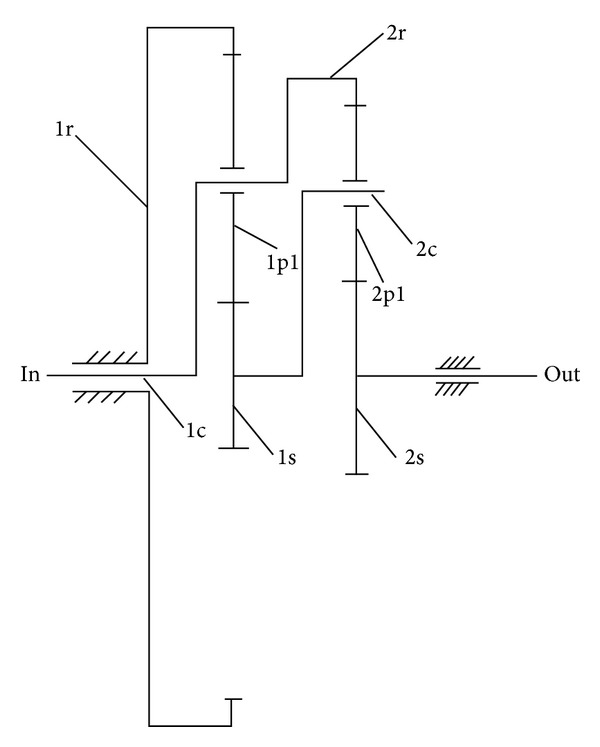
Kinematic scheme of load-split two-stage planetary gear.

The kinematic scheme of load-split two-stage planetary gear is shown in Figure 1, which is composed of closed planetary gear train and differential planetary gear train. Former basic units 1c (planetary carrier) and 1s (sun gear) are connected to units 2r (inner ring) and 2c (planetary carrier) of differential gear train, respectively. Therefore, load split is realized by first-stage and second-stage gear bearing input torque simultaneously. In Figure 1, 1r, 1p1, 1s, and 1c are inner ring, planetary gear, sun gear, and planetary carrier of first-stage planetary gear train, respectively, while 2r, 2p1, 2s, and 2c represent corresponding units of second-stage planetary gear train.

### 2.2. Speed of Each Unit of First-Stage Planetary Gear Train

The relationship between rotational speed of sun gear and that of planetary carrier and inner ring of first-stage planetary gear train is shown in

(1) $$\begin{array}{c} n_{1\text{s}}=i_{1\text{s}1\text{c}}^{1\text{r}}n_{1\text{c}}+i_{1\text{s}1\text{r}}^{1\text{c}}n_{1\text{r}}. \end{array}$$

Equation (2.2) is obtained according to general relationship of relative gear ratio among each unit in planetary gear train principle and transmission type and characteristic parameter of first-stage planetary gear train:

(2.2) $$\begin{array}{c} i_{1\text{s}1\text{c}}^{1\text{r}}=1-i_{1\text{s}1\text{r}}^{1\text{c}},\\ i_{1\text{s}1\text{r}}^{1\text{c}}=-\frac{Z_{1\text{r}}}{Z_{1\text{s}}},\\ \frac{Z_{1\text{r}}}{Z_{1\text{s}}}=\lambda_{1},\\ n_{1\text{r}}=0. \end{array}$$

We can come to (3) by (1) and (2.2):

(3) $$\begin{array}{c} n_{1\text{s}}=\left(1+\lambda_{1}\right)n_{1\text{c}}. \end{array}$$

The relationship between rotational speed of planetary gear and that of planetary carrier and inner ring is expressed as (4), according to relationship of relative rotational speed among each unit in the first-stage planetary gear train:

(4) $$\begin{array}{c} n_{1\text{p}}=i_{1\text{p}1\text{c}}^{1\text{r}}n_{1\text{c}}+i_{1\text{p}1\text{r}}^{1\text{c}}n_{1\text{r}}. \end{array}$$

Similar to (2.2), (5) is obtained as follows:

(5) $$\begin{array}{c} i_{1\text{p}1\text{c}}^{1\text{r}}=1-i_{1\text{p}1\text{r}}^{1\text{c}},\\ i_{1\text{p}1\text{r}}^{1\text{c}}=\frac{Z_{1\text{r}}}{Z_{1\text{p}}},\\ Z_{1\text{p}}=\frac{Z_{1\text{r}}-Z_{1\text{s}}}{2},\\ \frac{Z_{1\text{r}}}{Z_{1\text{s}}}=\lambda_{1},\\ n_{1\text{r}}=0. \end{array}$$

Thus, (6) can be obtained using (5) and (4):

(6) $$\begin{array}{c} n_{1\text{p}}=\left(1-\frac{2\lambda_{1}}{\lambda_{1}-1}\right)n_{1\text{c}}, \end{array}$$

where *λ*_1_ is the characteristic parameter of planetary gear train and *λ*_1_ = *Z*_1r_/*Z*_1s_. *Z*_1r_, *Z*_1s_, and *Z*_1p_ are tooth number of inner ring, sun gear, and planetary gear, respectively. *n*_1*j*_  (*j* = c, s, r, p) and *i*_1*a*1*b*_^1*x*^ (*a* = c, s, r, p; *b* = c, s, r, p; *x* = c, s, r, p) represent the rotational speed and relative gear ratio of each unit of first-stage planetary gear train, respectively.

### 2.3. Speed of Each Unit of Second-Stage Planetary Gear Train

The relationship between the rotational speed of sun gear train and that of planetary carrier and inner ring of second-stage planetary gear is expressed as follows:

(7) $$\begin{array}{c} n_{2\text{s}}=i_{2\text{s}2\text{c}}^{2\text{r}}n_{2\text{c}}+i_{2\text{s}2\text{r}}^{2\text{c}}n_{2\text{r}}. \end{array}$$

Equation (8) can be obtained according to the transmission characteristic of basic unit of second-stage planetary gear train:

(8) $$\begin{array}{c} i_{2\text{s}2\text{c}}^{2\text{r}}n_{2\text{c}}=\frac{n_{2\text{s}}^{2\text{r}}}{n_{2\text{c}}^{2\text{r}}}n_{2\text{c}}^{2\text{r}}=n_{2\text{s}}^{2\text{r}},\\ i_{2\text{s}2\text{r}}^{2\text{c}}n_{2\text{r}}=\frac{n_{2\text{s}}^{2\text{c}}}{n_{2\text{r}}^{2\text{c}}}n_{2\text{r}}^{2\text{c}}=n_{2\text{s}}^{2\text{c}}. \end{array}$$

Using (7) and (8) gives

(9) $$\begin{array}{c} n_{2\text{s}}=n_{2\text{s}}^{2\text{r}}+n_{2\text{s}}^{2\text{c}}. \end{array}$$

Equations (10) and (11) are obtained by the relative movement relationship of planetary gear train's units, when inner ring and planetary carrier of second-stage planetary gear train are fixed, respectively:

(10) $$\begin{array}{c} n_{2\text{r}}=0,\\ n_{2\text{s}}^{2\text{r}}=\frac{n_{2\text{c}}}{i_{2\text{c}2\text{s}}^{2\text{r}}},\\ i_{2\text{c}2\text{s}}^{2\text{r}}=\frac{1}{1-i_{2\text{s}2\text{r}}^{2\text{c}}},\\ i_{2\text{s}2\text{r}}^{2\text{c}}=-\frac{Z_{2\text{r}}}{Z_{2\text{s}}},\\ \frac{Z_{2\text{r}}}{Z_{2\text{s}}}=\lambda_{2}, \end{array}$$

(11) $$\begin{array}{c} n_{2\text{c}}=0,\\ n_{2\text{s}}^{2\text{c}}=\frac{n_{2\text{r}}}{i_{2\text{r}2\text{s}}^{2\text{c}}},\\ i_{2\text{r}2\text{s}}^{2\text{c}}=-\frac{Z_{2\text{s}}}{Z_{2\text{r}}},\\ \frac{Z_{2\text{r}}}{Z_{2\text{s}}}=\lambda_{2}. \end{array}$$

By connecting (10) and (11) to (9), the relationship of rotational speed between input and output units of second-stage planetary gear train is obtained as follows:

(12) $$\begin{array}{c} n_{2\text{s}}=\left(1+\lambda_{2}\right)n_{2\text{c}}-\lambda_{2}n_{2\text{r}}. \end{array}$$

And the relationship between rotational speeds of planetary gear is expressed as follows:

(13) $$\begin{array}{c} n_{2\text{p}}=i_{1\text{p}2\text{c}}^{2\text{r}}n_{2\text{c}}+i_{2\text{p}2\text{r}}^{2\text{c}}n_{2\text{r}}. \end{array}$$

Similar to (2.2), (14) can be obtained as follows:

(14) $$\begin{array}{c} i_{2\text{p}2\text{c}}^{2\text{r}}=1-i_{2\text{p}2\text{r}}^{2\text{c}},\\ i_{2\text{p}2\text{r}}^{2\text{c}}=\frac{Z_{2\text{r}}}{Z_{2\text{p}}},\\ Z_{2\text{p}}=\frac{Z_{2\text{r}}-Z_{2\text{s}}}{2},\\ \frac{Z_{2\text{r}}}{Z_{2\text{s}}}=\lambda_{2}. \end{array}$$

Substitution of (14) into (13) yields

(15) $$\begin{array}{c} n_{2\text{p}}=\frac{-\lambda_{2}-1}{\lambda_{2}-1}n_{2\text{c}}+\frac{2\lambda_{2}}{\lambda_{2}-1}n_{2\text{r}}. \end{array}$$

Considering the scheme of Figure 1, (16) can be given as follows:

(16) $$\begin{array}{c} n_{2\text{r}}=n_{1\text{c}},\\ n_{2\text{c}}=n_{1\text{s}}. \end{array}$$

Substitution of (16) and (3) into (15) gives

(17) $$\begin{array}{c} n_{2\text{p}}=\frac{2\lambda_{2}-\left(\lambda_{2}+1\right)\left(1+\lambda_{1}\right)}{\lambda_{2}-1}n_{1\text{c}}, \end{array}$$

where *λ*_2_ represents the characteristic parameter of planetary line of second-stage planetary gear train and *λ*_2_ = *Z*_2r_/*Z*_2s_. *Z*_2r_, *Z*_2s_, and *Z*_2p_ stand for tooth number of inner ring, sun gear, and planetary gear of second planetary gear train, respectively. *n*_2*j*_  (*j* = c, s, r, p) is the rotational speed of each unit of second-stage planetary gear train, and *i*_2*a*2*b*_^2*x*^ (*a* = c, s, r, p; *b* = c, s, r, p; *x* = c, s, r, p) is the relative gear ratio of corresponding unit.

### 2.4. Transmission Ratio of the Planetary Gear Train

The expressions of input and output rotational speed of load-split two-stage planetary gear train are given by substitution of (16) and (3) into (12), as follows:

(18) $$\begin{array}{c} n_{2\text{s}}=\left[\left(1+\lambda_{2}\right)\left(1+\lambda_{1}\right)-\lambda_{2}\right]n_{1\text{c}}. \end{array}$$

Thus, transmission ratio formula of load-split two-stage planetary gear train is obtained as

(19) $$\begin{array}{c} i_{2\text{s}1\text{c}}=\frac{n_{2\text{s}}}{n_{1\text{c}}}=\left(1+\lambda_{1}\right)\left(1+\lambda_{2}\right)-\lambda_{2}. \end{array}$$

General transmission ratio in Figure 1 is related to characteristic parameters of the planetary gear trains*λ*_1_ and *λ*_2_. Too small values of *Z*_1r_ and *Z*_2r_ result in undersizing of the system and decreasing of bearing capacity. Values of characteristic parameters have to be reasonable. Recommended interval of *λ*_1_ and *λ*_2_ is [3, 8].

**Figure 2:**
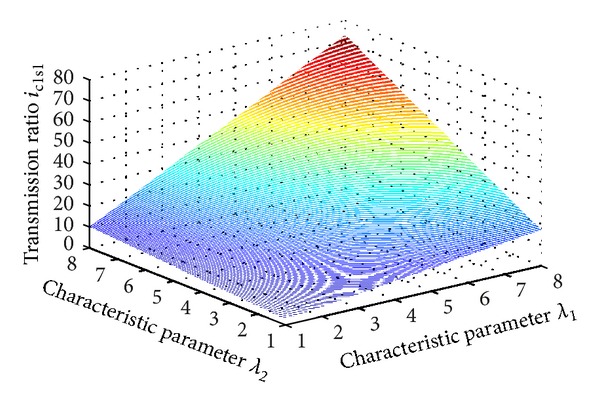
Relationship between transmission ratio and characteristic parameters of planetary gear train.

Relationship between transmission ratio and characteristic parameters in load-split two-stage planetary gear train is shown in Figure 2. Transmission ratio rises with increasing values of characteristic parameters of the planetary gear train, and maximum transmission ratio in the interval of [3, 8] is 73.

## 3. Dynamic Model

### 3.1. Model of the Multiple-Stage Transmission System

**Figure 3:**
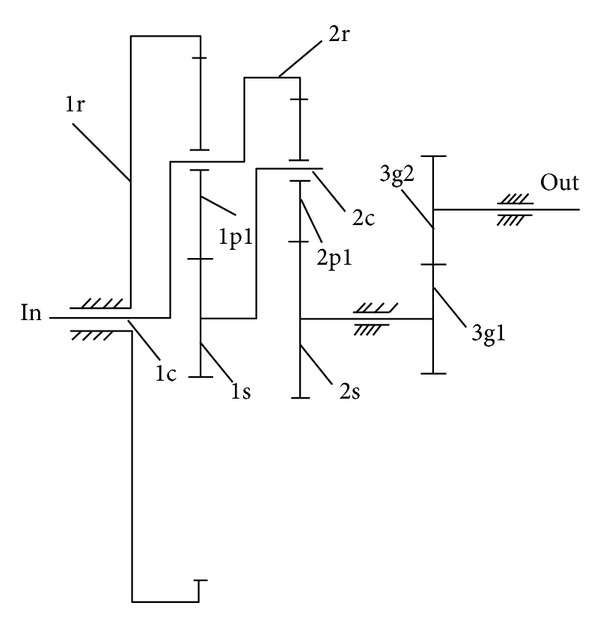
Transmission system of load-split multiple-stage planetary gear train.

A multiple-stage gear train composed of a two-stage planetary gear train and a one-stage parallel axis gear is shown in Figure 3. 3g1 and 3g2 in Figure 3 stand for pinion gear and driven gear of parallel axis.

**Figure 4:**
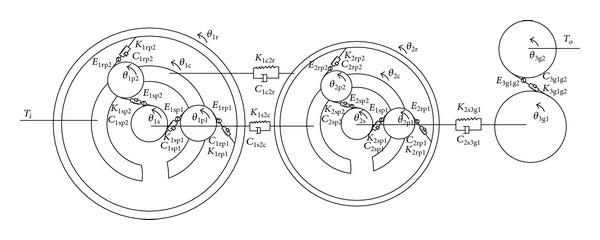
Dynamic model of load-split multiple-stage planetary gear train.

**Figure 5:**
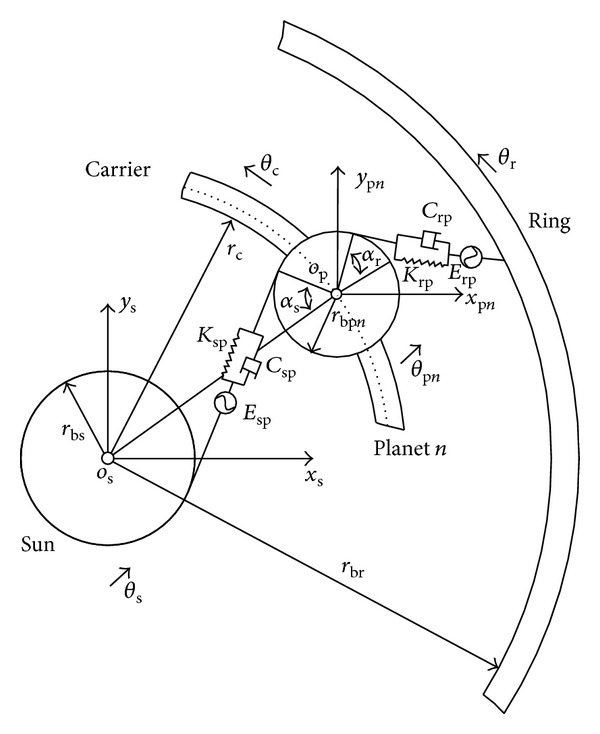
Torsional model of single-stage planetary gear.

Dynamic model of Figure 3 is shown in Figure 4 based on lumped parameter theory. Since the first-stage planetary gear train and the second-stage planetary gear train have the same basic structure, they can be represented by the single stage purely torsional model shown in Figure 5.

The linear displacements of all members of the multistage transmission system are shown as follows:

(20) $$\begin{array}{c} u_{1\text{c}}=r_{1\text{c}}\theta_{1\text{c}},\;\;u_{2\text{c}}=r_{2\text{c}}\theta_{2\text{c}},\\ u_{1\text{s}}=r_{1\text{bs}}\theta_{1\text{s}},\;\;u_{2\text{s}}=r_{2\text{bs}}\theta_{2\text{s}},\\ u_{1\text{r}}=r_{1\text{br}}\theta_{1\text{r}},\;\;u_{2\text{r}}=r_{2\text{br}}\theta_{2\text{r}},\\ u_{1\text{p}j}=r_{1\text{bp}j}\theta_{1\text{p}j},\;\;u_{2\text{p}j}=r_{2\text{bp}j}\theta_{2\text{p}j},\\ u_{3\text{g}1}=r_{3\text{bg}1}\theta_{3\text{g}1},\;\;u_{3\text{g}2}=r_{3\text{bg}2}\theta_{3\text{g}2}. \end{array}$$

Generalized masses of all members of the multistage transmission system are shown as follows:

(21) $$\begin{array}{c} m_{1\text{c}}=\frac{I_{1\text{ce}}}{r_{1\text{c}}^{2}},\;\;m_{2\text{c}}=\frac{I_{2\text{ce}}}{r_{2\text{c}}^{2}},\;\;m_{1\text{s}}=\frac{I_{1\text{s}}}{r_{1\text{bs}}^{2}},\\ m_{2\text{s}}=\frac{I_{2\text{s}}}{r_{2\text{bs}}^{2}},\;\;m_{1\text{r}}=\frac{I_{1\text{r}}}{r_{1\text{br}}^{2}},\;\;m_{2\text{r}}=\frac{I_{2\text{r}}}{r_{2\text{br}}^{2}},\\ m_{1\text{p}j}=\frac{I_{1\text{p}j}}{r_{1\text{bp}j}^{2}},\;\;m_{2\text{p}j}=\frac{I_{2\text{p}j}}{r_{2\text{bp}j}^{2}},\;\;m_{3\text{g}1}=\frac{I_{3\text{g}1}}{r_{3\text{bg}1}^{2}},\\ m_{3\text{g}2}=\frac{I_{3\text{g}2}}{r_{3\text{bg}2}^{2}}. \end{array}$$

### 3.2. Dynamic Equation of the Multistage Transmission System

The interaction force between sun gear and the *j*th planetary gear of the first-stage planetary gear train along the line of action is given as follows:

(22) $$\begin{array}{c} F_{1\text{sp}j}=K_{1\text{sp}j}X_{1\text{sp}j}+C_{1\text{sp}j}{\dot{X}}_{1\text{sp}j},\\ X_{1\text{sp}j}=u_{1\text{s}}+u_{1\text{p}j}-\cos\alpha_{1\text{s}}u_{1\text{c}}-E_{1\text{sp}j},\\ {\dot{X}}_{1\text{sp}j}={\dot{u}}_{1\text{s}}+{\dot{u}}_{1\text{p}j}-\cos\alpha_{1\text{s}}{\dot{u}}_{1\text{c}}-{\dot{E}}_{1\text{sp}j}. \end{array}$$

The interaction force between the inner ring and the *j*th planet gear of the first-stage planetary gear train along the line of action can be expressed as follows:

(23) $$\begin{array}{c} F_{1\text{rp}j}=K_{1\text{rp}j}X_{1\text{rp}j}+C_{1\text{rp}j}{\dot{X}}_{1\text{rp}j},\\ X_{1\text{rp}j}=u_{1\text{p}j}-u_{1\text{r}}+\cos\alpha_{1\text{r}}u_{1\text{c}}-E_{1\text{rp}j},\\ {\dot{X}}_{1\text{rp}j}={\dot{u}}_{1\text{p}j}-{\dot{u}}_{1\text{r}}+\cos\alpha_{1\text{r}}{\dot{u}}_{1\text{c}}-{\dot{E}}_{1\text{rp}j}. \end{array}$$

The interaction force between sun gear and the *j*th planetary gear of the second-stage planetary gear train along the line of action is

(24) $$\begin{array}{c} F_{2\text{sp}j}=K_{2\text{sp}j}X_{2\text{sp}j}+C_{2\text{sp}j}{\dot{X}}_{2\text{sp}j},\\ X_{2\text{sp}j}=u_{2\text{s}}+u_{2\text{p}j}-\cos\alpha_{2\text{s}}u_{2\text{c}}-E_{2\text{sp}j},\\ {\dot{X}}_{2\text{sp}j}={\dot{u}}_{2\text{s}}+{\dot{u}}_{2\text{p}j}-\cos\alpha_{2\text{s}}{\dot{u}}_{2\text{c}}-{\dot{E}}_{2\text{sp}j}. \end{array}$$

The interaction force between the inner ring and the *j*th planet gear of the second-stage planetary gear train along the line of action can be expressed as follows:

(25) $$\begin{array}{c} F_{2\text{rp}j}=K_{2\text{rp}j}X_{2\text{rp}j}+C_{2\text{rp}j}{\dot{X}}_{2\text{rp}j},\\ X_{2\text{rp}j}=u_{2\text{p}j}-u_{2\text{r}}+\cos\alpha_{2\text{r}}u_{2\text{c}}-E_{2\text{rp}j},\\ {\dot{X}}_{2\text{rp}j}={\dot{u}}_{2\text{p}j}-{\dot{u}}_{2\text{r}}+\cos\alpha_{2\text{r}}{\dot{u}}_{2\text{c}}-{\dot{E}}_{2\text{rp}j}. \end{array}$$

The interaction force between the pinion gear and driven gear of the third-stage parallel axis gear along the line of action can be expressed as follows:

(26) $$\begin{array}{c} F_{3\text{g}1\text{g}2}=K_{3\text{g}1\text{g}2}X_{3\text{g}1\text{g}2}+C_{3\text{g}1\text{g}2}{\dot{X}}_{3\text{g}1\text{g}2},\\ X_{3\text{g}1\text{g}2}=u_{3\text{g}1}+u_{3\text{g}2}-E_{3\text{g}1\text{g}2},\\ {\dot{X}}_{3\text{g}1\text{g}2}={\dot{u}}_{3\text{g}1}+{\dot{u}}_{3\text{g}2}-{\dot{E}}_{3\text{g}1\text{g}2}. \end{array}$$

Fix the inner ring of the first-stage planetary gear train, and take the number of planetary gears of the planetary gear train as 3; namely, 1*N* = 2*N* = 3. According to the planetary mechanism modeling methods in [13], dynamic equation of the multistage transmission system shown in Figure 4 can be built, as shown in

(27) m1cu¨1c−cosα1s∑j=11NF1spj+cosα1r∑j=11NF1rpj +K1c2rr1c(u1cr1c−u2rr2br)+C1c2rr1c(u˙1cr1c−u˙2rr2br)=Tir1c,m1su¨1s+∑j=11NF1spj+K1s2cr1bs(u1sr1bs−u2cr2c) +C1s2cr1bs(u˙1sr1bs−u˙2cr2c)=0,m1p1u¨1p1+F1sp1+F1rp1=0,m1p2u¨1p2+F1sp2+F1rp2=0,⋮m1p1Nu¨1p1N+F1sp1N+F1rp1N=0,m2cu¨2c−cosα2s∑j=12NF2spj+cosα2r∑j=12NF2rpj −K1s2cr2c(u1sr1bs−u2cr2c)−C1s2cr2c(u˙1sr1bs−u˙2cr2c)=0,m2su¨2s+∑j=12NF2spj+K2s3g1r2bs(u2sr2bs−u3g1r3bg1) +C2s3g1r2bs(u˙2sr2bs−u˙3g1r3bg1)=0,m2p1u¨2p1+F2sp1+F2rp1=0,m2p2u¨2p2+F2sp2+F2rp2=0,⋮m2p2Nu¨2p2N+F2sp2N+F2rp2N=0,m2ru¨2r−∑j=12NF2rpj−K1c2rr2br(u1cr1c−u2rr2br) −C1c2rr2br(u˙1cr1c−u˙2rr2br)=0,m3g1u¨3g1+F3g1g2−K2s3g1r3bg1(u2sr2bs−u3g1r3bg1) −C2s3g1r3bg1(u˙2sr2bs−u˙3g1r3bg1)=0,m3g2u¨3g2+F3g1g2+Tor3bg2=0.

The equations of the dynamic model are given in the matrix form as

(28) $$\begin{array}{c} M\ddot{u}+C\dot{u}+Ku=F, \end{array}$$

where the displacement vector, the mass matrix, the damping matrix, the stiffness matrix, and the load vector are given, respectively, as

(29) $$\begin{array}{c} u={\left[u_{1\text{c}},u_{1\text{s}},u_{1\text{p}1},u_{1\text{p}2},u_{1\text{p}3},u_{2\text{c}},u_{2\text{s}},u_{2\text{p}1},u_{2\text{p}2},u_{2\text{p}3},u_{2\text{r}},u_{3\text{g}1},u_{3\text{g}2}\right]}^{T},\\ M=\mathrm{diag}\left(m_{1\text{c}},m_{1\text{s}},m_{1\text{p}1},m_{1\text{p}2},m_{1\text{p}3},m_{2\text{c}},m_{2\text{s}},m_{2\text{p}1},m_{2\text{p}2},m_{2\text{p}3},m_{2\text{r}},m_{3\text{g}1},m_{3\text{g}2}\right),\\ C=\left[\begin{array}{ccccc} \frac{C_{1\text{c}2\text{r}}}{r_{1\text{c}}^{2}}+\cos\alpha_{1\text{s}}^{2}\sum_{j=1}^{3}C_{1\text{sp}j}+\cos\alpha_{1\text{r}}^{2}\sum_{j=1}^{3}C_{1\text{rp}j} & -\cos\alpha_{1\text{s}}\sum_{j=1}^{3}C_{1\text{sp}j} & C_{1\text{rp}1}\cos\alpha_{1\text{r}}-C_{1\text{sp}1}\cos\alpha_{1\text{s}} & C_{1\text{rp}2}\cos\alpha_{1\text{r}}-C_{1\text{sp}2}\cos\alpha_{1\text{s}} & \cdots \\ & -\cos\alpha_{1\text{s}}\sum_{j=1}^{3}C_{1\text{sp}j} & \frac{C_{1\text{s}2\text{c}}}{r_{1\text{bs}}^{2}}+\sum_{j=1}^{3}C_{1\text{sp}j} & C_{1\text{sp}1} & \cdots \\ & & C_{1\text{sp}1}+C_{1\text{rp}1} & 0 & \cdots \\ & & & C_{1\text{sp}2}+C_{1\text{rp}2} & \cdots \\ \text{symmetric} & & & & \ddots \end{array}\right],\\ K=\left[\begin{array}{ccccc} \frac{K_{1\text{c}2\text{r}}}{r_{1\text{c}}^{2}}+\cos\alpha_{1\text{s}}^{2}\sum_{j=1}^{3}K_{1\text{sp}j}+\cos\alpha_{1\text{r}}^{2}\sum_{j=1}^{3}K_{1\text{rp}j} & -\cos\alpha_{1\text{s}}\sum_{j=1}^{3}K_{1\text{sp}j} & K_{1\text{rp}1}\cos\alpha_{1\text{r}}-K_{1\text{sp}1}\cos\alpha_{1\text{s}} & K_{1\text{rp}2}\cos\alpha_{1\text{r}}-K_{1\text{s}p2}\cos\alpha_{1\text{s}} & \cdots \\ & -\cos\alpha_{1\text{s}}\sum_{j=1}^{3}K_{1\text{sp}j} & \frac{K_{1\text{s}2\text{c}}}{r_{1\text{bs}}^{2}}+\sum_{j=1}^{3}K_{1\text{sp}j} & K_{1\text{sp}1} & \cdots \\ & & K_{1\text{sp}1}+K_{1\text{rp}1} & 0 & \cdots \\ & & & K_{1\text{sp}2}+K_{1\text{rp}2} & \cdots \\ \text{symmetric} & & & & \ddots \end{array}\right],\\ F=\left[\begin{array}{c} \frac{T_{i}}{r_{1\text{c}}}-\cos\alpha_{1\text{s}}\left(\sum_{j=1}^{3}C_{1\text{sp}j}{\dot{E}}_{1\text{sp}j}+\sum_{j=1}^{3}K_{1\text{sp}j}E_{1\text{sp}j}\right)+\cos\alpha_{1\text{r}}\left(\sum_{j=1}^{3}C_{1\text{rp}j}{\dot{E}}_{1r\text{p}j}+\sum_{j=1}^{3}K_{1\text{rp}j}E_{1\text{rp}j}\right)\\ \sum_{j=1}^{3}C_{1\text{sp}j}{\dot{E}}_{1\text{sp}j}+\sum_{j=1}^{3}K_{1\text{sp}j}E_{1\text{sp}j}\\ C_{1\text{sp}1}{\dot{E}}_{1s\text{p}1}+K_{1\text{sp}1}E_{1\text{sp}1}+C_{1\text{rp}1}{\dot{E}}_{1\text{rp}1}+K_{1\text{rp}1}E_{1\text{rp}1}\\ C_{1\text{sp}2}{\dot{E}}_{1\text{sp}2}+K_{1\text{sp}2}E_{1\text{sp}2}+C_{1\text{rp}2}{\dot{E}}_{1\text{rp}2}+K_{1\text{rp}2}E_{1\text{rp}2}\\ C_{1\text{sp}3}{\dot{E}}_{1\text{sp}3}+K_{1\text{sp}3}E_{1\text{sp}3}+C_{1\text{rp}3}{\dot{E}}_{1\text{rp}3}+K_{1\text{rp}3}E_{1\text{rp}3}\\ \cos\alpha_{2\text{r}}(\sum_{j=1}^{3}K_{2\text{rp}j}E_{2\text{rp}j}+\sum_{j=1}^{3}C_{2\text{rp}j}{\dot{E}}_{2\text{rp}j})-\cos\alpha_{2\text{s}}(\sum_{j=1}^{3}K_{2\text{sp}j}E_{2\text{sp}j}+\sum_{j=1}^{3}C_{2\text{sp}j}{\dot{E}}_{2\text{sp}j})\\ \vdots \end{array}\right]. \end{array}$$

## 4. Load Sharing Characteristic of Load-Split Multiple-Stage Planetary Gear Train

### 4.1. Calculation of Load Sharing Coefficient

Use numerical integration method for solving the dynamic equation (28) of the system, obtain the responses to displacement and velocity of the system, and substitute the responses into (22)–(25), and then obtain the engaging forces  *F*_1sp*j*_, *F*_1rp*j*_, *F*_2sp*j*_, and *F*_2rp*j*_. Make *D*_1sp*jk*s_ and *D*_1sp*jk*r_, respectively, represent the load sharing coefficients of the internal and external meshing of all gear pairs of the first-stage planetary gear train and *D*_2sp*ik*s_ and *D*_2sp*ik*r_ as those of the internal and external meshing of all gear pairs of the second-stage planetary gear train; then load sharing coefficients are expressed as

(30) $$\begin{array}{c} D_{1\text{sp}jk\text{s}}=\frac{1N{\left(F_{1\text{sp}jk\text{s}}\right)}_{\max}}{\sum_{j=1}^{1N}{\left(F_{1\text{sp}jk\text{s}}\right)}_{\max}},\\ D_{1\text{rp}jk\text{r}}=\frac{1N{\left(F_{1\text{rp}jk\text{r}}\right)}_{\max}}{\sum_{j=1}^{1N}{\left(F_{1\text{rp}jk\text{r}}\right)}_{\max}},\\ D_{2\text{sp}ik\text{s}}=\frac{2N{\left(F_{2\text{sp}jk\text{s}}\right)}_{\max}}{\sum_{i=1}^{2N}{\left(F_{2\text{sp}jk\text{s}}\right)}_{\max}},\\ D_{2\text{rp}ik\text{r}}=\frac{2N{\left(F_{2\text{rp}jk\text{r}}\right)}_{\max}}{\sum_{i=1}^{2N}{\left(F_{2\text{rp}jk\text{r}}\right)}_{\max}}, \end{array}$$

where *k*s, *k*r are meshing cycle numbers for internal and external meshing of the planetary gear pair.

When *d*_1sp*j*_ and *d*_1rp*j*_ are used to stand for load sharing coefficient of internal and external meshing of each first-stage gear and *d*_2sp*j*_ and *d*_2rp*j*_ for that of each second-stage gear in system period, respectively, the expression can be given as follows:

(31) $$\begin{array}{c} d_{1\text{sp}j}={\left|D_{1\text{sp}jk\text{s}}-1\right|}_{\max}+1,\\ d_{1\text{rp}j}={\left|D_{1\text{rp}jk\text{r}}-1\right|}_{\max}+1,\\ d_{2\text{sp}j}={\left|D_{2\text{sp}jk\text{s}}-1\right|}_{\max}+1,\\ d_{2\text{rp}j}={\left|D_{2\text{rp}jk\text{r}}-1\right|}_{\max}+1. \end{array}$$

The paper analyzes the transmission system as shown in Figure 4. The basic parameters of the transmission system are shown in Tables 1 and 2, and other parameters can be determined by [21]. Substitute the relevant parameters of the system into (28) for solution. Use (30) and (31) to obtain the load sharing coefficients of the transmission system.

### 4.2. Influence of Mesh Error on Load Sharing Coefficient of the System

**Figure 6:**
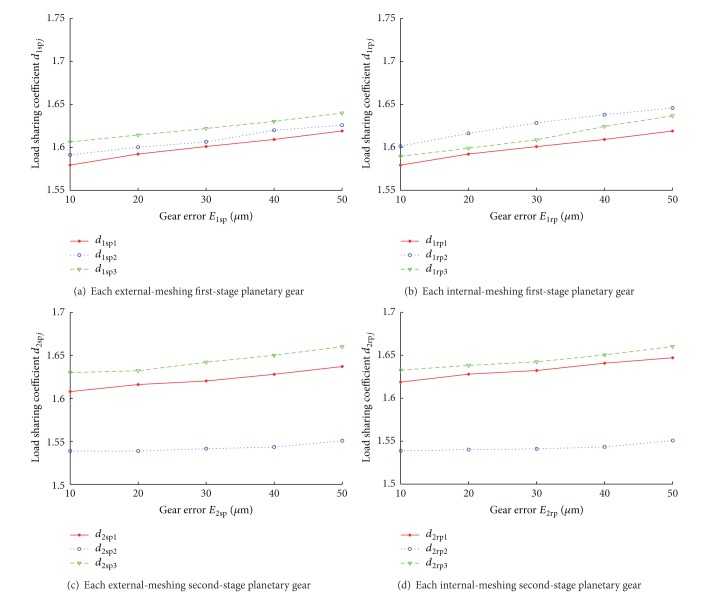
Load-sharing coefficient curves of each planetary gear with different mesh errors.

Load sharing property of planetary gear train is significantly affected by manufacturing error, installation error, and eccentric error, which cannot be neglected in planetary gear train. Considering system's complexity, it is assumed that equivalent mesh error of each stage planetary gear at the direction of meshing line is equal, and values of 10, 20, 30, 40, and 50 *μ*m are given, respectively. Load sharing properties of multiple-stage gear train under these five conditions are studied. Relationships between load sharing coefficient curves of internal and external meshing of first-stage and second-stage, which are calculated according to (31), are drawn in Figure 6, with different mesh errors.

Results below can be concluded according to Figure 6.

1. Each load-sharing coefficient increases with increasing mesh error.
2. Load sharing coefficient of internal-meshing is different from that of external-meshing under different mesh errors. Maximum external-meshing and internal-meshing load sharing coefficients of first-stage planetary gear are 1.579 and 1.645, respectively, while those of second-stage planetary gear are 1.630 and 1.665, respectively.
3. Compared to the differences in change rate of each load sharing coefficient of second-stage planetary gear, that of first-stage planetary gear is more evident. The maximum difference in change rate of first-stage planetary gear is 0.101/50 *μ*m, while that of second-stage planetary gear is only 0.003/50 *μ*m.

### 4.3. Influence of Revolution Speed on Load Sharing Coefficient

To analyze the influence of revolution speed of the first-stage planetary gear on load sharing coefficient, the revolution speed is set as 5 r/min, 10 r/min, 15 r/min, 20 r/min, and 25 r/min, respectively. Equation (31) is used to calculate the load sharing coefficient under different conditions, and curves are obtained inFigure 7.

**Figure 7:**
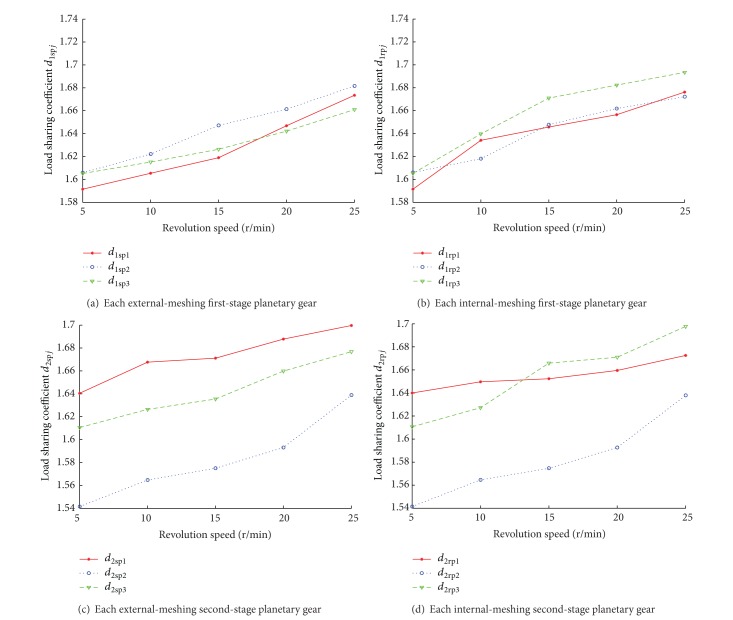
Load sharing coefficient curves of each planetary at different revolution speeds.

Influence of revolution speed on load-sharing coefficient can be concluded below, according to Figure 7.

1. Each load sharing coefficient increases with raising the revolution speed, which indicates that load sharing capacity of planetary gear train is weakened and vibration is aggravated with increasing revolution speed.
2. At the variation interval of revolution speed, the change rate difference of load-sharing coefficient between internal and external meshing of first-stage planetary gear train is significantly different; those of first-stage planetary gears 1, 2, and 3 are 1.77%, 0.84%, and 1.49%, respectively. Similar result can be concluded in second-stage planetary gear train, and change rate differences of 1.47%, 2.71%, and 2.76% of second-stage planetary gears 1, 2, and 3 are figured out, respectively.

## 5. Conclusion

1. The dynamic model is built to account for the dynamic behavior of multiple-stage planetary gear train used in wind driven generator. The model can provide useful guideline for the dynamic design of the multiple-stage planetary gear train of wind driven generator.
2. Each load-sharing coefficient of the first-stage planetary gear varies more than that of the second-stage planetary gear. At the same mesh error, second-stage internal-meshing load sharing coefficient is the largest, the first-stage internal-meshing load sharing coefficient is the second largest, and the first-stage external-meshing load sharing coefficient is the minimum.
3. Load sharing property is weakened and transmission system's vibration is aggravated with increasing revolution speed. At each interval of revolution speed, internal and external meshing load sharing coefficients of the second-stage planetary gear train vary more than those of the first-stage planetary gear train.

## Figures and Tables

**Table 1:** Primary parameters of planetary gear train.

Parameter	Carrier	Ring	Sun gear	Planetary gear
Pitch radius, *r*_1_ (mm)	468	726	210	258
Base circle, *r*_1*b*_ (mm)	—	682.22	197.34	242.44
Mass,*M*_1_ (kg)	2042.77	410.34	344.91	388.39
Moment of inertia, *J*_1_ (kg·m^2^)	462.55	226.57	7.62	16.25
Pressure angle, *α*_1_ (°)	—	20	20	20
Pitch radius, *r*_2_ (mm) *r*_2_/mm	345	550	140	205
Base circle, *r*_2*b*_ (mm)	—	516.83	131.56	192.64
Mass, *M*_2_ (kg)	1212.49	80.56	131.40	176.54
Moment of inertia, *J*_2_ (kg·m^2^)	151.75	25.65	1.29	5.12
Pressure angle, *α*_2_ (°)	—	20	20	20

**Table 2:** Primary parameters of parallel-shaft gears.

Parameter	Pinion gear	Driven gear
Pitch radius, *r*_3_ (mm)	292	100
Base circle, *r*_3*b*_ (mm)	274.39	93.97
Mass, *M*_3_ (kg)	208.22	24.14
Moment of inertia, *J*_3_ (kg·m^2^)	8.87	0.12
Pressure angle, *α*_3_ (°)	20	20

## Nomenclature

*θ*_*i*_: Angular displacement of *i*th member (*i* = s, p*n*, r; *n* = 1, 2, 3)
*r*_b*i*_: Gear base radii, *i* = s, p*n*, r; *n* = 1,2, 3
*r*_c_: Radius of the circle passing through planet centers
*r*_*i*c_: *i*th-stage radii of the circle passing through planet centers; *i* = 1,2
*α*_s_: Sun-planet engaging angle
*α*_r_: Ring-planet engaging angle
*iN*: Total number of planet sets for the *i*th-stage drive train; *i* = 1,2
*I*_1*j*_: Polar mass moment of inertia of *j*th member for 1st-stage drive train; *j* = c, s, p1, p2,…, p1*N*
*M*_1p_: Mass of 1st-stage planetary gear
*I*_1ce_: = *I*_1c_ + 1*NM*_1p_ *r*_1c_^2^
*I*_2*j*_: Polar mass moment of inertia of *j*th member for 2nd-stage drive train; *j* = c, s, p1, p2,…, p2*N*
*M*_2p_: Mass of 2nd-stage planetary gear
*I*_2ce_: = *I*_2c_ + 2*NM*_2p_ *r*_2c_^2^
*r*_*i*b*j*_: Gear base radii of *j*th member for *i*th-stage drive train; *i* = 1,2, 3; *j* = s, r, p1, p2,…, p*n*, g1, g2
*α*_*i*s_: Sun-planet engaging angle for *i*th-stage drive train; *i* = 1,2
*α*_*i*r_: Ring-planet engaging angle for *i*th-stage drive train; *i* = 1,2
*E*_sp_: Sun-planet mesh error
*E*_rp_: Ring-planet mesh error
*K*_sp_: Sun-planet mesh stiffness
*K*_rp_: Ring-planet mesh stiffness
*C*_sp_: Sun-planet mesh damping coefficient
*C*_rp_: Ring-planet mesh damping coefficient
*θ*_*ij*_: Angular displacement of *j*th member for *i*th-stage drive train; *i* = 1,2, 3; *j* = s, r, c, p1, p2,…, p*n*, g1, g2; *n* = 1,2, 3
*E*_*i*sp*j*_: Sun-planet mesh error of *j*th planet gear for *i*th-stage drive train; *i* = 1,2; *j* = 1,2, 3
*E*_*i*rp*j*_: Ring-planet mesh error of *j*th planet gear for *i*th-stage drive train; *i* = 1,2; *j* = 1,2, 3
*E*_3g1g2_: Mesh error of parallel-shaft gears
*K*_*i*sp*j*_: Sun-planet mesh stiffness of *j*th planet gear for *i*th-stage drive train; *i* = 1,2; *j* = 1,2, 3
*K*_*i*rp*j*_: Ring-planet mesh stiffness of *j*th planet gear for *i*th-stage drive train; *i* = 1,2; *j* = 1,2, 3
*K*_3g1g2_: Mesh stiffness of parallel-shaft gears
*K*_1s2c_: Torsional stiffness associated with 1st-stage sun and 2nd-stage carrier
*K*_1c2r_: Torsional stiffness associated with 1st-stage carrier and 2nd-stage ring
*K*_2s3g1_: Torsional stiffness associated with 2nd-stage sun and 3rd-stage gear
*C*_*i*sp*j*_: Sun-planet mesh damping coefficient of *j*th planet gear for *i*th-stage drive train; *i* = 1,2; *j* = 1,2, 3
*C*_*i*rp*j*_: Ring-planet mesh damping coefficient of *j*th planet gear for *i*th-stage drive train; *i* = 1,2; *j* = 1,2, 3
*C*_3g1g2_: Mesh damping coefficient of parallel-shaft gears
*C*_1s2c_: Torsional damping coefficient associated with 1st-stage sun and 2nd-stage carrier
*C*_1c2r_: Torsional damping coefficient associated with 1st-stage carrier and 2nd-stage ring
*C*_2s3g1_: Torsional damping coefficient associated with 2nd-stage sun and 3rd-stage gear
*T*_*i*_: Input torque
*T*_*o*_: Output torque.
